# Timing of treatment interruption among latently infected tuberculosis cases treated with a nine-month course of daily isoniazid: findings from a time to event analysis

**DOI:** 10.1186/s12889-019-7524-4

**Published:** 2019-09-03

**Authors:** Marie Nancy Séraphin, HsiaoChu Hsu, Helena J. Chapman, Joanne L. de Andrade Bezerra, Lori Johnston, Yang Yang, Michael Lauzardo

**Affiliations:** 1 Division of Infectious Diseases and Global Medicine, Department of Medicine, University of Florida, 2055 Mowry Road, Suite 250, Gainesville, FL 32611 USA; 2 Emerging Pathogens Institute, University of Florida, 2055 Mowry Road, Gainesville, FL 32610 USA; 3 Bureau of Communicable Diseases, Tuberculosis Control Section, Florida Department of Health, 4052 Bald Cypress Way, Tallahassee, FL 32399 USA; 4 Department of Biostatistics, College of Public Health and Health Professions, University of Florida, 2055 Mowry Road, suite 250, Gainesville, FL 32610 USA

**Keywords:** Latent tuberculosis infection, Prevention, Treatment outcomes

## Abstract

**Background:**

Treatment of latent tuberculosis infection (LTBI) in high-risk groups is an effective strategy for TB control and elimination in low incidence settings. A nine-month course of daily isoniazid (INH) has been the longest prescribed therapy; however, completion rates are suboptimal. We need data to guide TB program outreach efforts to optimize LTBI treatment completion rates.

**Methods:**

We pooled seven (2009–2015) years of LTBI treatment outcome data. We computed the probability of INH treatment disruption over time by patient demographic and clinical risk factors. We used log-rank tests and Cox proportional hazards models to assess the risk factors for treatment disruption.

**Results:**

We analyzed data from 12,495 persons with complete data on INH treatment initiation. Pediatric cases (0–17 years), recent contacts of active TB patients, and non-U.S.-born adults living in the United States ≤5 years represented 25.2, 13.0, and 59.2% of the study population, respectively. Overall, 48.4% failed to complete therapy. The median treatment duration was 306 days (95% CI: 297, 315). A significant drop in adherence could be observed around day 30 of treatment initiation. Indeed, by day 30 of treatment, 17.0% (95% CI: 16.4, 17.7) of patients had defaulted on therapy. Pediatric patients (HR = 0.83, 95% CI: 0.78, 0.89), recent contacts (HR = 0.74, 95% CI: 0.68, 0.81), patients with diabetes (HR = 0.77, 95% CI: 0.60, 0.98), and patients with HIV (HR = 0.39, 95% CI: 0.30, 0.51) had a lower risk of treatment default. However, black patients (HR = 1.57, 95% CI: 1.44, 1.70), Hispanic patients (HR = 1.54, 95% CI: 1.43, 1.66), and non-U.S.-born persons living in the United States ≤5 years (HR = 1.25, 95% CI: 1.18, 1.32) were significantly more likely to default on therapy.

**Conclusions:**

In this analysis of INH treatment outcome, we see high levels of treatment discontinuation. On average, patients defaulted on their prescribed nine-month daily INH therapy within 30 days of initiating treatment, and those at increased risk of progression to active disease were most likely to do so. We highlight the need to introduce patient-centered programs to increase treatment adherence in this population.

**Electronic supplementary material:**

The online version of this article (10.1186/s12889-019-7524-4) contains supplementary material, which is available to authorized users.

## Background

In low tuberculosis (TB) incidence countries like the United States (U.S.), early identification and treatment of persons with asymptomatic or latent *Mycobacterium tuberculosis* infection (LTBI) are fundamental for sustainable prevention and control efforts toward TB elimination (< 1 TB case / 1 million population) [1]. This indispensable approach is in light of an estimated one-third of the global population infected with *M. tuberculosis*, who face a 5 to 10% risk of progression to active TB disease during their lifespan [2]. By identifying populations at high-risk for TB disease – such as recent contacts, diabetics, or persons with immunosuppression [e.g., human immunodeficiency virus (HIV) / acquired immunodeficiency syndrome (AIDS), dialysis or organ transplant recipients, selected pharmacological agents] – targeted LTBI diagnostic testing and clinical evaluation can lead to the most effective treatment regimen, thus reducing risk of TB disease [3]. These concerted efforts for prompt LTBI diagnosis and effective treatment have the potential to reduce global TB incidence rates, curb transmission, and ultimately reach the targets of the World Health Organization’s End TB Strategy (2016–2035) [4]. In the U.S., TB incidence has decreased over the past two decades, leading to current incidence rates that lie above the TB elimination threshold of one TB case per million person per year [5]. In 2017, 9,093 new TB cases (2.9 cases / 100,000 population) were reported [6, 7]. The incidence among persons born outside of the U.S. was 14.6/100,100 population, compared to 1.0/100,000 among the U.S.-born population [6]. However, risk factors associated with TB reactivation in high-risk population groups, remain a significant challenge for continued TB elimination efforts. Previous studies concluded that an estimated 93% of foreign-born persons diagnosed with active TB disease were due to TB reactivation [8]. Hence, with 13 million U.S. residents estimated to have LTBI, this high-risk population group should be prioritized for diagnostic testing and prophylaxis treatment [9].

Isoniazid (INH) was the first anti-tuberculosis drug recommended for LTBI treatment, a decision supported by findings from several randomized clinical trials conducted in the 1960s [10, 11]. Although a prescribed nine-month INH regimen is preferred and considered more efficacious, a six-month INH regimen can be alternatively prescribed to improve patient adherence [12]. INH selectively inhibits the cytochrome P450 enzymes of *M. tuberculosis* and is noted for its narrow spectrum of action for mycobacteria species and high bactericidal activity [13]. Aside from the benefits of INH monotherapy for LTBI management, there are reported drawbacks such as extended treatment duration, additional health care costs, potential toxicities, and risk of INH monoresistance [14–16]. Serious adverse effects can contribute to patient treatment non-compliance and potential INH discontinuation, including hepatotoxicity risk estimated between 1 and 4%, occurring within the first few months after treatment initiation [17, 18]. Peripheral neuropathy—which can be prevented with vitamin B6 (pyridoxine) supplementation—as well as dermatitis and lupus-like syndrome are other INH adverse effects [19].

Acceptance and completion of LTBI treatment, based on the total number of administered doses taken, continue to be a hurdle in clinical practice. In many TB programs throughout the U.S., including Florida, the nine-month course of INH for LTBI therapy is self-administered. Patients are given enough drugs for a month and are required to go back to the health center for monthly evaluation of side effects and refills. Previous studies have reported several predictors of acceptance of LTBI treatment among patients, including an understanding of LTBI and their ability to transmit active TB disease to their close contacts, convenient scheduling for medical appointment and supportive staff, and low acculturation [20]. On the other hand, risk factors associated with LTBI treatment default or discontinuation have included severe side effects, unemployment, and limited social support [21, 22]. Non-adherence rates (< 80% of prescribed dose regimen) range from 31 to 53% [15, 17, 23–25]. However, limited data exist on the timing of treatment discontinuation, which are important to the design of interventions to increase overall adherence to therapy. In this study, we investigated the timing of INH treatment defaults and associated risk factors to guide TB program outreach efforts to meet the specific needs of diverse patient populations, optimize LTBI treatment completion, and reduce risk of TB reactivation.

## Methods

### Study population

Data from a LTBI treatment registry collected from 2009 to 2015 were pooled for these analyses. Persons with LTBI were diagnosed and locally managed by the 67 different county health departments in Florida, and the Florida Department of Health TB Control Program centrally pooled the data. LTBI diagnosis was based on either a positive tuberculosis skin test (TST) and/or interferon gamma release assay (IGRA) test followed by a medical evaluation and additional testing consisting of chest x-ray and, in some cases, an acid-fast sputum smear examination to rule out active TB disease. We included everyone initiated on INH in the analysis. Patients were prescribed self-administered INH 300 mg per kg body weight daily, for 9 months. Drugs were dispensed during scheduled monthly visits to the health department LTBI clinic. LTBI is not a reportable condition in Florida. As such, these data only capture people diagnosed and managed by county health departments’ TB clinics in Florida.

### Treatment outcomes and censoring

All treatment outcomes were available within the registry and recorded at the health department level by a provider involved in the care of the LTBI client. We determined the duration of treatment by calculating the number of days between the date of treatment initiation and the date of treatment termination. We excluded from the analyses patients with a treatment plan that was open for longer than 12 months as well as patients who completed therapy in less than 6 months. Based on treatment outcomes, patients were classified into five groups: treatment completed, loss to follow-up, chose to stop, adverse reactions, and dead. We treated loss to follow-up and chose to stop as observed default events. The other outcomes were right-censored. Data on patients’ gender, race, HIV status, diabetes status, and other immunosuppressive conditions collected at baseline were also included in the analyses to evaluate predictors for treatment disruption.

### Statistical analysis

We conducted all analyses using the Statistical Analysis System (SAS) version 9.4 and the R programming language version 3.6.0. We computed the probability of INH treatment disruption over time for each of the demographic and clinical risk factor variables and displayed those using Kaplan-Meier curves. To compare the time to treatment interruption for each level of our covariates, we used log-rank with log-log transformation for the pointwise confidence bounds. Statistical significance was assessed at the level of 0.05. We computed univariate and multivariable hazard ratio with the 95% confidence intervals (CI) of treatment disruption using Cox proportional hazards models. We visually assessed the assumption of proportionality of hazards for our Cox models by inspecting the group-based empirical cumulative hazard curves for extreme non-proportionality and none of the covariates included in the analyses violated this assumption (see Additional file 1: Figure S1). We used Efron correction to account for ties in the treatment disruption time.

## Results

**Fig. 1:**
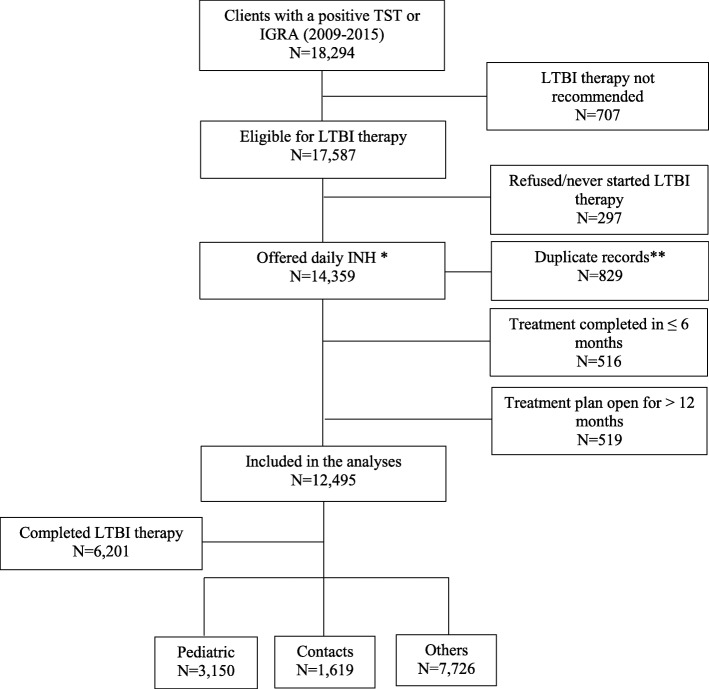
Flow diagram detailing selection of cases included in the analyses. TST indicates tuberculin skin test; IGRA indicates interferon gamma release assay; LTBI indicates latent tuberculosis infection; INH indicates Isoniazid. * Others were offered either three months of isoniazid and rifapentine (3HP) or four months of rifampin (4R) ** Includes 28 observations where all demographic and clinical data were the same but treatment outcome differed

Overall, 18,294 persons had a positive TST and/or IGRA test results with a diagnosis of LTBI over the study period (Fig. 1). Individuals who were not a candidate for LTBI therapy; i.e. those with a history of TB or LTBI treatment, or pregnant; were excluded from the analyses (*n* = 707). We excluded individuals who refused or never initiated therapy (*n* = 297). We also excluded persons who completed therapy in less than 6 months (*n* = 516), persons with duplicate case IDs (*n* = 829), and patients with a care plan that had been opened for longer than 12 months (*n* = 519). About half of the individuals in this group ultimately completed therapy (*n* = 333); however, not finishing within 12 months is more or less a type of default, which does not fit in the definition of default we focused on this study. In Additional file 1, we show a distribution of the treatment duration data by the different outcomes after (Additional file 1 Figure S2A) and before (Additional file 1Figure S2B). We noticed that the overall time distribution of the treatment outcomes did change. In addition, including these cases as a sensitivity analysis did not change the overall study findings (Additional file 1: Figure S3). This leads us to conclude that the excluded observations were more likely outliers and data recording errors that did not significantly bias the overall study sample.

Table 1 shows the characteristics of the LTBI patients included in our study, stratified by their treatment outcomes. Of the 12,495 patients, 25.2% were pediatric cases (0–17 years), 13.0% were recent contacts to an infectious TB case, and 61.8% were other adults, the majority of which were non-U.S.-born persons (60.4%). Overall, the majority of our study sample composed of non-U.S-born persons who had immigrated within the last 5 years (52.2%) and non-Hispanic patients (54.5%). The racial composition of the sample was 36.0% White, 34.1% Black/African American, and 7.5% Asian. About 2 % were HIV co-infected at the time of diagnosis, 1.3% were diabetic, and less than 1.0% had one or more other immunosuppressive conditions.

**Table 1:** Characteristics of persons with latent tuberculosis infection who initiated INH monotherapy and followed for twelve months

Predictors (*N* = 12,495)	Sample N (%)	Treatment Completed N (%)	Loss to Follow N (%)	Chose to Stop N (%)	Adverse Reaction N (%)	Died N (%)	*p*-value
		6201 (49.6)	3120 (25.0)	2928 (23.4)	237 (1.9)	9 (0.1)	
Type of Patients							
Pediatric Recent Contact Other Adults	3150 (25.2) 1619 (13.0) 7726 (61.8)	1639 (52.0) 901 (55.6) 3661 (47.4)	823 (26.1) 382 (23.6) 1915 (24.8)	635 (20.2) 310 (19.1) 1983 (25.7)	53 (1.7) 25 (1.5) 159 (2.1)	- 1 (0.1) 8 (0.1)	<.0001
Gender							
Female Male Missing	6076 (48.6) 6417 (51.4) 2 (0.0)	3090 (50.9) 3111 (48.5) -	1433 (23.6) 1685 (26.3) 2 (100.0)	1400 (23.0) 1528 (23.8) -	150 (2.5) 87 (1.4)	3 (0.0) 6 (0.1)	<.0001
Ethnicity							
Non-Hispanic Hispanic Unknown	6808 (54.5) 5513 (44.1) 174 (1.4)	3714 (54.5) 2394 (43.4) 93 (53.4)	1881 (27.6) 1189 (21.6) 50 (28.7)	1093 (16.0) 1807 (32.8) 28 (16.1)	114 (1.7) 120 (2.2) 3 (1.7)	6 (0.1) 3 (0.0) -	<.0001
Race							
White Black Asian Other	4496 (36.0) 4261 (34.1) 932 (7.5) 2806 (22.5)	2027 (45.1) 2035 (47.8) 650 (69.7) 1489 (53.1)	1004 (45.1) 1328 (47.8) 194 (20.8) 594 (21.2)	1350 (30.0) 833 (19.5) 80 (8.6) 665 (23.7)	110 (2.4) 63 (1.5) 7 (0.7) 57 (2.0)	5 (0.1) 2 (0.0) 1 (0.1) 1 (0.0)	<.0001
Birth Origin							
U.S.-born Non-U.S.-born, ≤ 5 Years Non-U.S.-born, > 5 Years	5097 (40.8) 6517 (52.2) 881 (7.0)	2780 (54.5) 2998 (46.0) 423 (48.0)	1325 (26.0) 1591 (24.4) 204 (23.2)	890 (17.5) 1802 (27.6) 236 (26.8)	96 (1.9) 124 (1.9) 17 (1.9)	6 (0.1) 2 (0.0) 1 (0.1)	<.0001
HIV Co-infected							
No Yes	12,275 (98.2) 220 (1.8)	6038 (49.2) 163 (74.1)	3087 (25.1) 33 (15.0)	2907 (23.7) 21 (9.5)	236 (1.9) 1 (0.4)	7 (0.1) 2 (0.9)	<.0001
Diabetic							
No Yes	12,512 (98.2) 170 (1.3)	6099 (49.5) 102 (60.4)	3079 (25.0) 41 (24.3)	2906 (23.6) 22 (13.0)	234 (1.9) 3 (1.8)	8 (0.1) 1 (0.6)	0.0010
Other Immunosuppressive Conditions*							
No Yes	12,395 (99.2) 100 (0.8)	6136 (49.5) 65 (65.0)	3104 (25.0) 16 (16.0)	2913 (23.5) 15 (15.0)	235 (1.9) 2 (2.0)	7 (0.1) 2 (2.0)	<.0001

Notes: * includes individuals with hematologic disorders (*n* = 6), chronic renal failure (*n* = 21), non-HIV related immunosuppressive conditions (*n* = 48), and those on corticosteroid therapy (n = 21)

Overall, 48.4% of the sample failed to complete their nine-month INH treatment regimen; about 2 % (*n* = 237) experienced an adverse reaction to the treatment and 0.1% (*n* = 9) died. While a quarter of the cases were lost to follow up, another quarter (23.4%) chose to stop therapy (Table 1). The median treatment duration was 306 days (95% CI: 297, 315). Median INH treatment duration was 325 days (95% CI: 313–335) days for pediatric cases, 344 (322–356) days for recent contacts, and 276 (95% CI: 258–295) days for other adults.

**Fig. 2:**
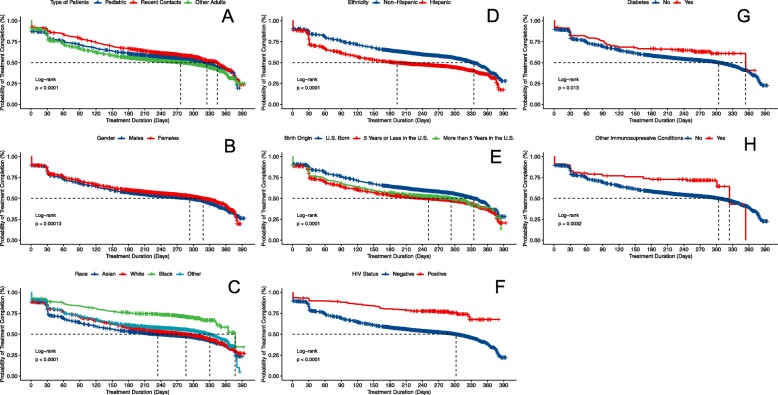
Kaplan-Meier plots of the duration of INH treatment. Plots are stratified by type of patients (A), demographic (B - E) and clinical risk factors (F - H)

The baseline cumulative hazard of treatment completion for the whole sample (Additional file 1 Figure S1, panel A) and the sample stratified by patient type (panel B) are presented in Additional file 1. A significant drop in adherence can be observed in the first 30 days of treatment initiation. Indeed, 17.0% (95% CI: 16.4–17.7) of patients who initiated therapy had discontinued therapy by day 30. The nine-month INH therapy is dispensed at monthly intervals during scheduled visits to the LTBI clinic, suggesting that the first 30 days after treatment initiation are the most vulnerable time point for treatment default and loss to follow-up. We evaluated the probabilities of non-disruption in the first 4 weeks of therapy. Figure 2 shows the probabilities of staying on the nine-month INH therapy, stratified by each of the risk factors evaluated in this study. On average, males had a shorter treatment duration of 293 (95% CI: 271, 304) days, compared to 318 (95% CI: 308, 333) days among females. The duration of treatment was also shorter for non-U.S.-born, Hispanic, Whites, HIV-negative, patients without diabetes, and patients without an immunosuppressive condition. At 30 days after treatment initiation, 17.9% (95% CI: 17.0, 18.9) of males compared to 16.1% (95% CI: 15.2, 17.0) of females had interrupted their nine-month INH therapy. Similarly, at day 30 of therapy, 21.9% (95% CI: 20.8, 23.0) of Hispanic patients had interrupted their nine-month INH therapy, compared to 13.2% (95% CI: 12.4, 14.0) of non-Hispanic patients.

The cumulative incidence of INH treatment interruption for Asian patients at day 30 of therapy was 9.7% (95% CI: 7.9, 11.7), compared to 20.5% (95% CI: 19.3, 21.7) among White patients, 16.1% (95% CI: 15.0, 17.2) among Black patients, and 15.4% (95% CI: 14.1, 16.8) among the other racial groups. By the first month of INH therapy, 20.1% (95% CI: 19.2, 21.1) of patients who had recently immigrated to the U.S. (≤5 years) had discontinued treatment, compared to 12.9% (95% CI: 12.0, 21.1) in U.S.-born, and 17.9% (95% CI: 15.5, 20.6) in those who immigrated to the U.S. > 5 years. On the other hand, persons co-infected with HIV [8.2% (95% CI: 5.2, 12.7) versus 17.2% (95% CI: 16.5, 17.9)], those with diabetes [15.4% (95% CI: 10.8, 21.8) versus 17.1% (95% CI: 16.4, 17.8)], and one or more other immunosuppressive conditions [14.0% (95% CI: 8.5, 22.5) versus 17.4% (95% CI: 16.4, 17.7)] had a lower cumulative incidence of treatment interruption at 4 weeks.

The univariate and multivariable hazard ratios for treatment disruption are presented in Table 2. Pediatric patients (HR = 0.83, 95% CI: 0.78, 0.89) and recent contacts (HR = 0.74, 95% CI: 0.68, 0.81) had a significantly lower risk of treatment interruption compared to other adults. The risk of treatment interruption was also lower for female patients (HR = 0.90, 95% CI: 0.85, 0.94), Asian patients (HR = 0.77, 95% CI: 0.67, 0.89), those co-infected with HIV (HR = 0.39, 95% CI: 0.30, 0.51), diabetic patients (HR = 0.77, 95% CI: 0.60, 0.98), and those with one or more immunosuppressive conditions (HR = 0.65, 95% CI: 0.46, 0.93). On the other hand, the risk was significantly higher for White patients (HR = 1.26, 95% CI: 1.18, 1.36) and Black patients (HR = 1.57, 95% CI: 1.44, 1.70) compared to other racial groups; for non-U.S.-born individuals who immigrated ≤5 years (HR = 1.25, 95% CI: 1.18, 1.32) compared to U.S.-born patients; and Hispanic patients (HR = 1.54, 95% CI: 1.43, 1.66) compared to non-Hispanic patients.

**Table 2:** Risk Factors for isoniazid treatment interruptions among latent tuberculosis cases followed for 12 months

Predictors	Unadjusted HR (95% CI)	Adjusted HR (95% CI)
Type of Patient		
Pediatric	0.87 (0.82, 0.93)	0.83 (0.78, 0.89)
Recent Contact	0.74 (0.69, 0.81)	0.74 (0.68, 0.81)
Other Adults	1.00	1.00
Gender		
Male	1.00	1.00
Female	0.91 (0.86, 0.95)	0.90 (0.85, 0.94)
Ethnicity		
Non-Hispanic	1.00	1.00
Hispanic	1.46 (1.38, 1.53)	1.54 (1.43, 1.66)
Race		
White	1.28 (1.19, 1.37)	1.26 (1.18, 1.36)
Black	1.14 (1.06, 1.22)	1.57 (1.44, 1.70)
Asian	0.58 (0.51, 0.66)	0.77 (0.67, 0.89)
Other	1.00	1.00
Birth Origin		
U.S.-born	1.00	1.00
Non-U.S.-born, ≤ 5 Years	1.34 (1.27, 1.41)	1.25 (1.18, 1.32)
Non-U.S.-born, > 5 Years	1.24 (1.12, 1.37)	1.16 (1.04, 1.29)
HIV Co-infected		
No	1.00	1.00
Yes	0.41 (0.31, 0.53)	0.39 (0.30, 0.51)
Diabetic		
No	1.00	1.00
Yes	0.73 (0.57, 0.94)	0.77 (0.60, 0.98)
Other Immunosuppressive Conditions		
No	1.00	1.00
Yes	0.59 (0.42, 0.85)	0.65 (0.46, 0.93)

Notes: *HR* hazard ratio; * *p*-values are based on the Cox proportional hazards models

## Discussion

It is estimated that 1.7 billion of the world population has LTBI, with a 5−10% lifetime risk of developing active disease [26, 27]. This risk is especially elevated for high-risk groups, such as children < 5 years of age, recent immigrants from high TB burden countries, diabetics, those co-infected with HIV, or those with other non-HIV immunosuppressive conditions [28]. In addition, LTBI reactivation in these groups can drive low TB transmission rates in communities [14, 29]. In the U.S., about 11 million people have LTBI [30]. To have the greatest impact on TB elimination in low incidence settings, studies using mathematical modeling have shown that high-risk groups should be targeted for LTBI diagnosis and treatment [29]. Beyond recent contacts, children < 5 years old, the immunocompromised, and foreign-born persons from high TB burden countries represent a priority group for TB programs in the U.S. [6]. Foreign-born persons, especially recent arrivals (within ≤5 years of immigration) from high incidence countries, are at high risk of TB reactivation. Studies have shown that most of the disease burden is concentrated within the first 5 years of immigration [31, 32]. INH is currently the drug of choice for most programs due to its tolerability and established effectiveness [33]. However, INH treatment default is very common and a number of studies have reported risk factors for defaulting, including loss to follow up [34, 35]. In this study, we show that foreign-born, ethnic, and racial minorities are at increased risk of defaulting treatment within the first month of the nine-month INH therapy. This indicates that a significant number of LTBI cases who accept LTBI treatment and are sent home with their first monthly dose likely never initiate LTBI therapy.

In addition to 9 months of daily INH, other approved LTBI therapy options include 3 months of weekly INH and Rifapentine (3HP) by directly observed therapy (DOT), and 4 months of daily, self-administered Rifampin (4R) [36]. The shorter treatment options have better compliance rates compared to nine-month of daily INH [37]. Nevertheless, non-adherence remains an issue in high-risk groups [34]. Under actual use scenario, 85% of patients complete 3HP by DOT and this completion rate is comparable for 4R [25]. New clinical trials are underway to evaluate the safety and efficacy of even shorter regimens [38]. Beyond shortening duration of LTBI therapy, we should address and evaluate the various reasons why patients discontinue or never initiate LTBI therapy.

Perhaps it is possible to improve LTBI treatment adherence and completion if programs incorporate evidence from the long-term management of other infectious diseases, such as HIV. Some of the facilitators to antiretroviral therapy (ART) adherence outlined in the literature could potentially be applied to LTBI screening and treatment. At the heart of successful ART initiation is assuring that patients accept their diagnosis, are motivated and ready to initiate treatment, and have social support [39]. Operational research has also highlighted the importance of designing a treatment regimen that fits into a patient’s daily schedule and using reminder tools such as pill organizers [39]. Perhaps it is even more important to establish patient-provider rapport that is positive and free of judgment [39]. In our study, we observed that persons at high-risk of LTBI reactivation (i.e. young children, recent contacts, and those with immuno-suppressive conditions including HIV and diabetes) had a longer time to treatment default, compared to patients without these clinical risk factors. This may suggest an advantage to integrate effective risk communication within the overall LTBI care management. For example, close monitoring and counseling throughout the duration of LTBI therapy could enhance patient-provider communication and inform the best way to communicate the risk of progression to active disease to patients who otherwise do not perceive themselves at risk.

Our study has some limitations. We observed that treatment default occurred around day 30 of the nine-month daily INH regimen. In reality, the treatment default could have happened earlier; however, we were unable to measure the timing of treatment discontinuation more accurately. Indeed, INH monotherapy for LTBI is self-administered, and clients are not required to bring back bottles for pill count, which prevents the assessment of the percentage of prescribed pills that were taken before treatment default. A client is recorded as lost to follow-up by the health unit only after they have missed their scheduled monthly clinic visit, which involves a medication refill and an evaluation for medication side effects. In addition, the co-morbidity risk factor data evaluated in this study, such as HIV co-infection and diabetes, are self-reported and likely suffer from underreporting bias. To evaluate the completeness of the data, we compare the prevalence of these two co-morbidities in a TB registry collected 2009–2015. Active TB is reportable by statute in the State of Florida [40]. In addition, the TB program reports all cases to the Centers for Disease Control and Prevention (CDC), using a report of verified case of tuberculosis (RVCT) [41]. In that population of 4,911 TB cases, HIV prevalence is 12.7% and diabetes prevalence is 11.7%. These results suggest that these two co-morbidity risk factors are underreported in the LTBI dataset used in this study. In addition, it is possible that LTBI clients with HIV and diabetes are managed in the community by their primary care providers and are not reported to their county health departments because LTBI is not a reportable condition in Florida. Finally, these data were collected as part of LTBI treatment management and surveillance and not for research. As such, many important variables that could influence treatment default, such as patients’ social support and patient-provider communication, were not measured. Future studies should take these factors and the limitations listed above into consideration.

## Conclusion

In this analysis of INH treatment initiation data collected over 7 years in Florida, we see high levels of treatment discontinuation, with 17% of clients who initiated the nine-month daily regimen defaulting on therapy within the first 30 days. Because of data limitations, there are some uncertainties around the exact default time; however, it is possible these clients defaulted a lot sooner than recorded. Perhaps a universal approach to LTBI prevention efforts is not the solution to reach TB elimination by 2050. Instead, we could look to enhance LTBI treatment adherence through innovative and holistic approaches that consider patients’ understanding and awareness of LTBI, communicate the importance of treatment adherence, and remove social determinants of health that negatively influence health-seeking behaviors [42]. At the national level, strengthening connections between primary care providers and local health departments is crucial to accessing high-risk population groups and elevating LTBI treatment completion rates [43, 44]. Globally, with continued migration from high TB burden to low TB burden countries alike, international policies should reinforce systematic LTBI screening and management to support the End TB Strategy TB targets and TB elimination by 2050 [4, 45].

## Additional file

Additional file 1: Supplementary Figures S1 – S3. (PDF 561 kb)

## Data Availability

The data analyzed during the current study are not publicly available because they contain private health information (PHI) but are available from the corresponding author on reasonable request.

## Abbreviations

3HP: Three-month Isoniazid and Rifapentine
4R: Four-month rifampicin
ART: Antiretroviral therapy
CDC: Centers for Disease Prevention and Control
CI: Confidence Interval
DOT: Directly Observed Therapy
FDOH: Florida Department of Health
HIV/AIDS: Human Immunodeficiency Virus/Acquired Immunodeficiency Syndrome
HR: Hazard Ratio
IGRA: Interferon gamma release assay
INH: Isoniazid
IRB: Institutional Review Board
LTBI: Latent tuberculosis infection
PHI: Protected Health Information
RVCT: Report of Verified Case of Tuberculosis
TB: Tuberculosis
TST: Tuberculin skin test
U.S.: United States
