# Supplementary material for: Timing of treatment interruption among latently infected tuberculosis cases treated with a nine-month course of daily isoniazid: findings from a time to event analysis
Source: BMC Public Health. 2019 Sep 3;19:1214. doi: 10.1186/s12889-019-7524-4 (PMC6724263; doi:10.1186/s12889-019-7524-4)
Supplement: Supplementary file 1 — Supplementary Figures S1 – S3. (PDF 561 kb) [file 12889_2019_7524_MOESM1_ESM.pdf]

Supplementary Figures for "Timing of treatment interruption among latently infected tuberculosis cases treated with a nine-month course of daily isoniazid: findings from a time to event analysis"

Marie Nancy Séraphin[1, 2*]; HsiaoChu Hsu[1, 2]; Helena J. Chapman[1, 2]; Joanne Chen-Tse Li[1, 2]; Lori Johnston[3]; Yang Yang[2, 4] and Michael Lauzardo[1, 2]

Authors' information:

[1]Division of Infectious Diseases and Global Medicine, College of Medicine, University of Florida, 2055 Mowry Road, suite 250, Gainesville, FL 32610

[2]Emerging Pathogens Institute, University of Florida, 2055 Mowry Road, suite 250, Gainesville, FL 32610

[3]Bureau of Communicable Diseases, Tuberculosis Control Section, Florida Department of Health, 4052 Bald Cypress Way, Tallahassee, FL 32399

[4]Department of Biostatistics, College of Public Health and Health Professions, University of Florida, 2055 Mowry Road, suite 250, Gainesville, FL 32610*Corresponding Author

***** Corresponding author: nseraphin@ufl.edu; +1 352-273-9584

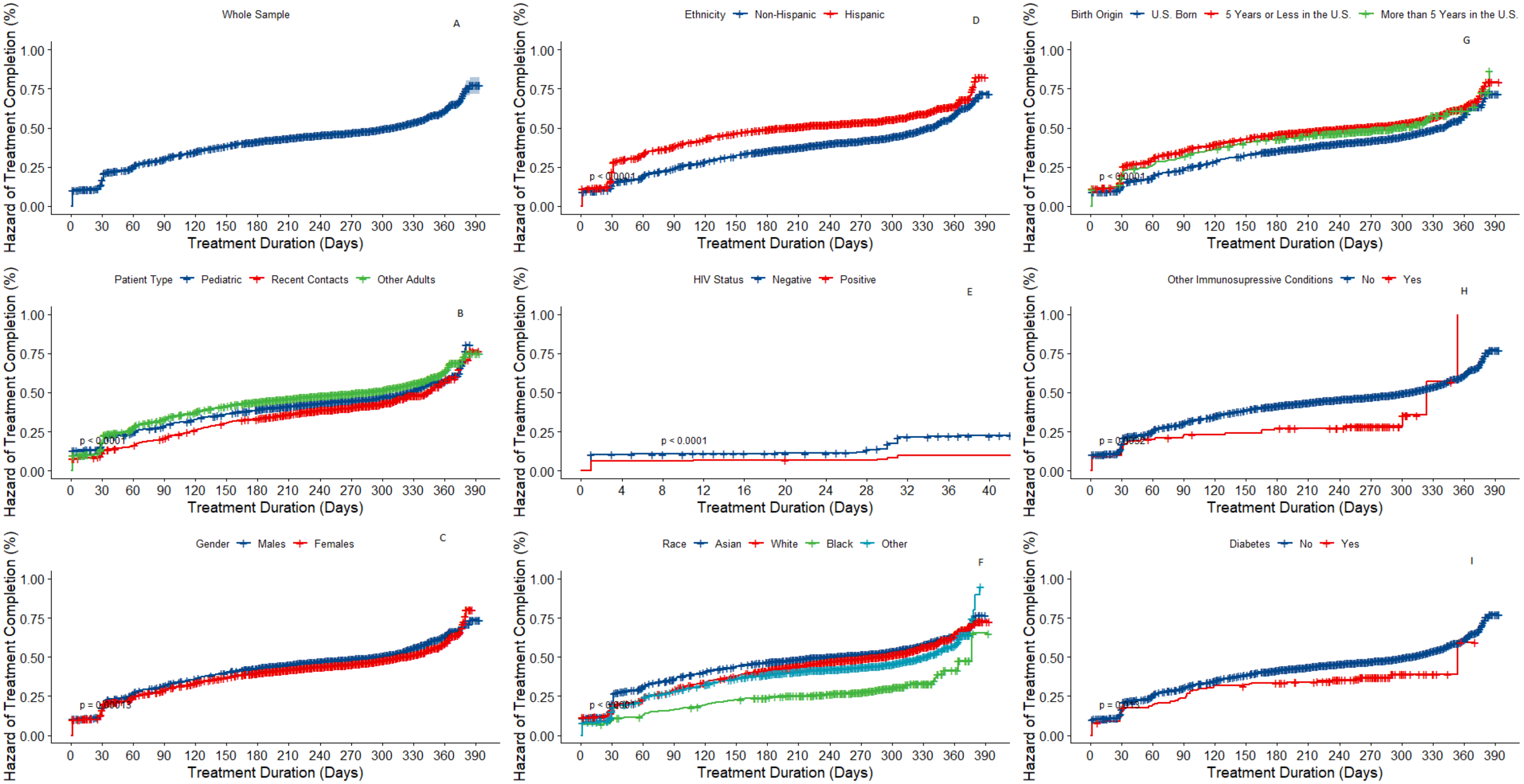

Figure S1. Cumulative hazard plots for the sample (A), by patient types (B) and risk factors (C – I).

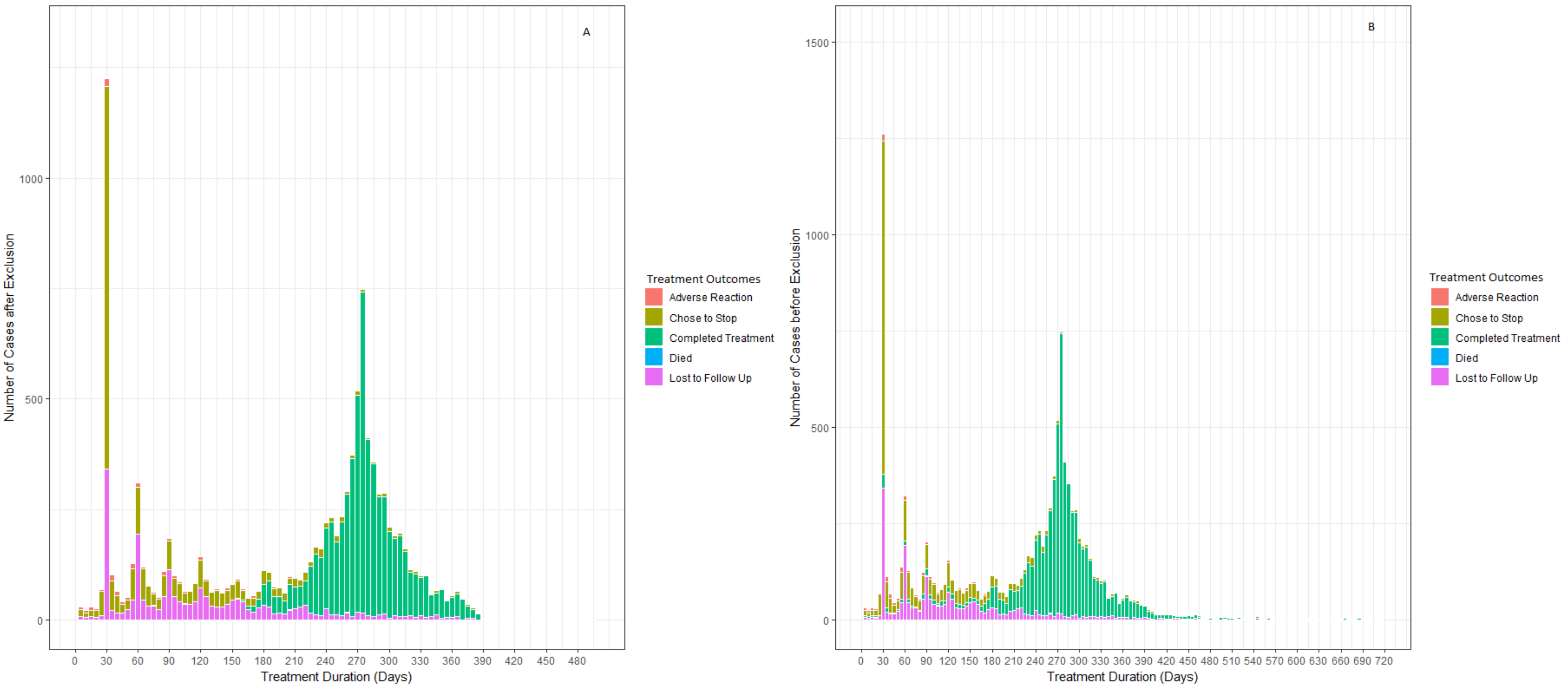

Figure S2. Treatment time distribution after (A) and before (B) exclusion of clients recorded as on treatment for longer than 12 months and those who completed therapy in less than six months.

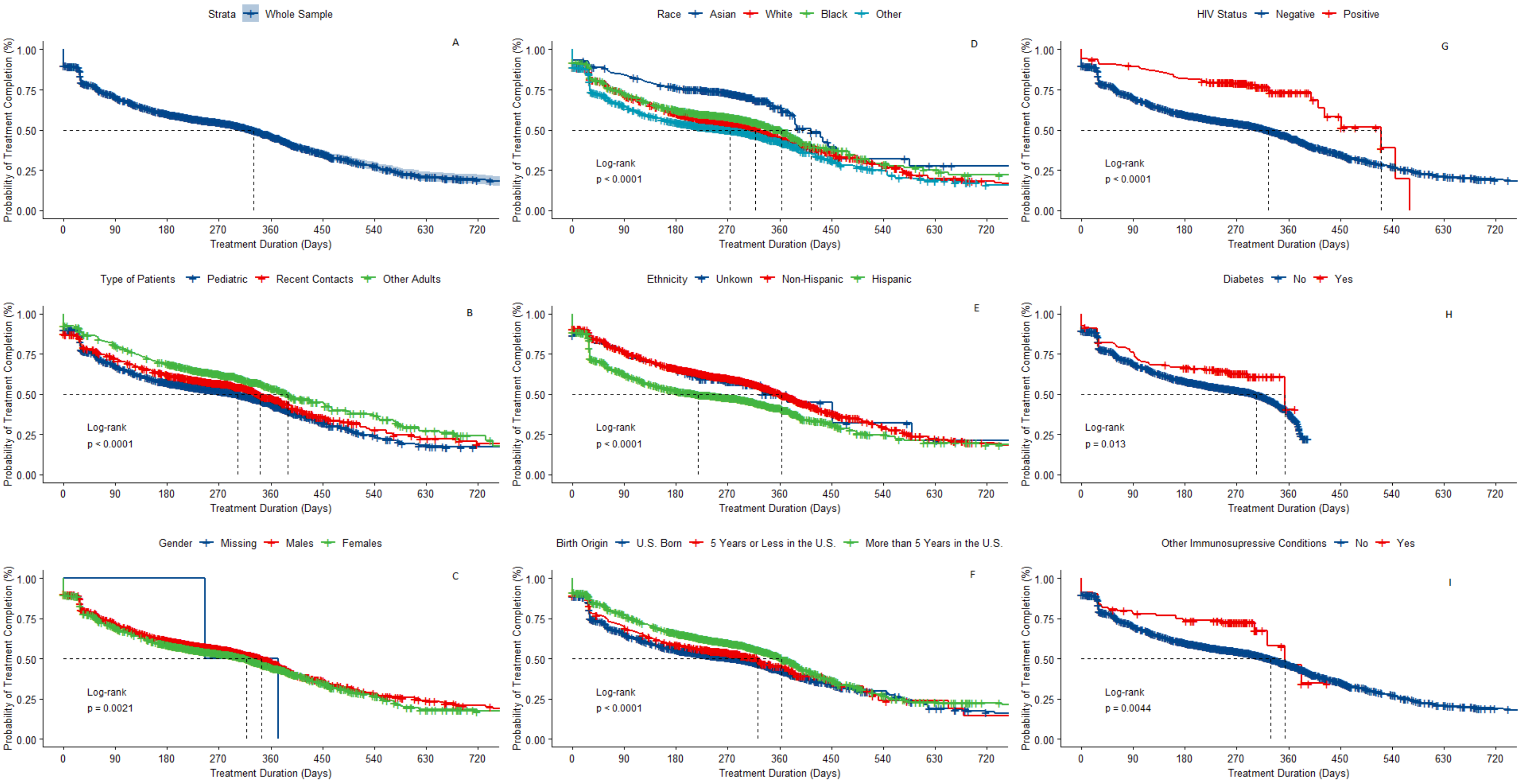

Figure S3. Kaplan-Meier plots of the duration of isoniazid (INH) treatment before exclusion of clients recorded as on treatment for longer than 12 months. Plots are stratified by demographic and clinical risk factors.
